# Panzootic HPAIV H5 and risks to novel mammalian hosts

**DOI:** 10.1038/s44298-024-00039-z

**Published:** 2024-05-29

**Authors:** E. M. Abdelwhab, Martin Beer

**Affiliations:** 1 Institute of Molecular Virology and Cell Biology, Friedrich-Loeffler-Institut, Südufer 10, Greifswald-Insel Riems, 17493 Germany; 2 Institute of Diagnostic Virology, Friedrich-Loeffler-Institut, Südufer 10, Greifswald-Insel Riems, 17493 Germany

## Abstract

The H5 subtype of highly pathogenic avian influenza viruses represents a significant challenge to animal and human health. H5 clade 2.3.4.4b viruses have experienced an unprecedented global spread, coupled with remarkable genetic plasticity for adaptation in birds and mammals. Although human infections remain very limited, the establishment in wild, marine, and farmed animals, including recently dairy cattle, is of concern. The role of mammalian hosts as intermediaries for zoonotic or even pandemic influenza A viruses should not be underestimated. In order to mitigate the zoonotic risk and be adequately prepared, it is essential to understand and monitor the dynamics of HPAIV H5 at the avian-mammal interface.

## Diversity of avian influenza viruses

Avian influenza viruses (AIVs) have their reservoir in wild birds, especially waterfowl^1^. However, they also continue to cause losses in the poultry industry worldwide, affecting global meat and egg production, threatening wild bird biodiversity and posing zoonotic risks^1^. The influenza A virus (IAV) genome is segmented into eight distinct, negative-stranded, RNA gene segments. The error-prone nature of the IAV polymerase, which is responsible for viral replication, allows for constant change and adaptation^1^, which is highly advantageous for viral evolution. When two different IAVs infect a host cell, gene segments can be swapped (i.e., reassortment or antigenic shift) to theoretically generate 254 novel genotypes^2^. Moreover, stepwise mutations acquired spontaneously during viral replication, may also allow the virus to adapt to new hosts (i.e. antigenic drift). To date, AIVs have been classified into 16 hemagglutinin (HA; H1-H16) and 9 neuraminidase (NA; N1-N9) subtypes based on variation in these surface glycoproteins. The type of HA and NA define the respective subtype (e.g. H5N1) with 144 possible HxNy combinations.

## Early history of HPAIV H5N1

The first reports of HPAIV in poultry date back to 1878 by *Edoardo Perroncito* in Italy^3^. Over a century later, in 1996, HPAIV of subtype H5N1 was identified in domestic geese in the Guangdong province (Gs/Gd), China^4^. In 1997, HPAIV H5N1 Gs/Gd was detected in poultry markets in Hong Kong, and 6 out of 18 infected humans died, drawing attention to the zoonotic risk of this virus group. Subsequently, HPAIV H5N1 Gs/Gd was controlled in commercial poultry, but it continued to circulate in the wild bird reservoir. In 2002, Gs/Gd-like viruses jumped again from wild birds to poultry and back to wild birds in the Qinghai Lake region^5^. The endemicity of this virus in wild birds and poultry, and the continuous exchange between poultry and wild birds resulted in successful evolution and diversification into numerous phylogenetic clades^6^. Since 2005, HPAIVs H5N1 Gs/Gd have been transmitted in several waves across regions and continents, primarily by migratory wild birds^7^.

## Currently circulating H5N1 of clade 2.3.4.4b

**Fig. 1:**
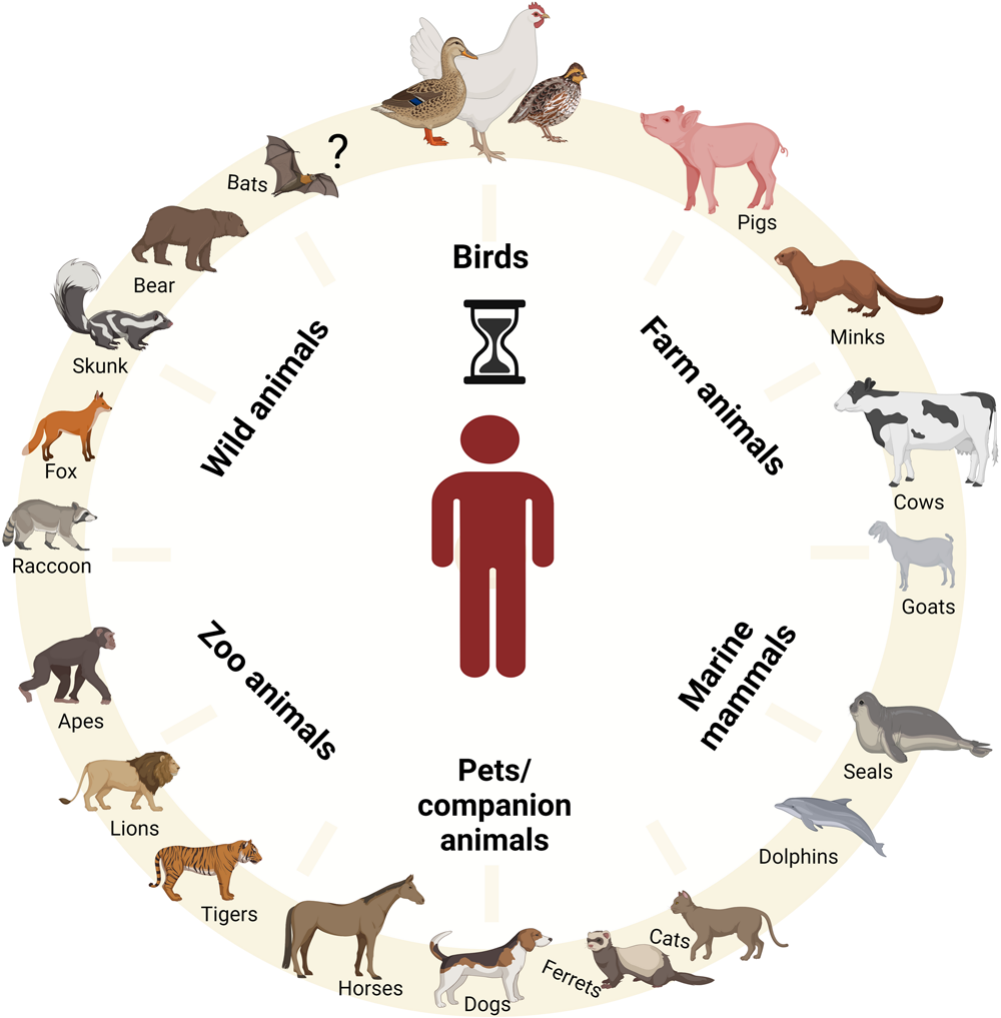
Potential hosts for the genesis of a zoonotic avian influenza virus. Mammals infected with the current AIV H5N1 clade 2.3.4.4b present a significant risk for the emergence of zoonotic viruses. Human infections have been documented in a dairy cow farm worker. Human-adaptation markers have been identified in multiple isolates from mammals, raising concerns about the potential role of these animals as intermediate hosts for the emergence of zoonotic AIV H5N1. The figure was created with BioRender.com.

Since 2022, HPAIVs of subtype H5 have become deeply entrenched in poultry and wild birds^7,8^. The subsequent panzootic clade 2.3.4.4b H5-viruses have a very wide range of avian hosts^9^ and show numerous spill-over infections to wild carnivores, farmed fur animals such as mink, fox and raccoon dogs, and marine mammals such as sea lions^10^ (Fig. 1). The unique characteristics of HPAIV H5 clade 2.3.4.4b demand a fundamental rethink of our understanding of these pathogens. Europe is now a new center for HPAIV H5 in wild birds, and reassortment with other European AIV is underway^11^. HPAIV H5 has been transmitted from Europe to Africa and via Iceland to North and South America. This has resulted in high overall mortality in naïve bird populations and mammals including on mainland Antarctica^12–14^. It is of great concern, that HPAIV H5 has infected well over 150 different bird species and more than 50 mammalian species (Fig. 1)^15–17^. In addition, HPAIV H5N1 of clade 2.3.4.4b has a remarkable genetic elasticity for reassortment with other AIVs resulting in a wide range of highly diverse H5N1, H5N2, H5N3, H5N4, H5N5, H5N6 and H5N8 viruses^18,19^. Efficient reassortment has resulted in well over 50 different genotypes^8^, with the HA segment as the stable element. This poses a significant challenge to the study of the epidemiology, pathobiology and genetic characteristics of these viruses^20^.

## HPAIV H5N1 in dairy cattle

**Fig. 2:**
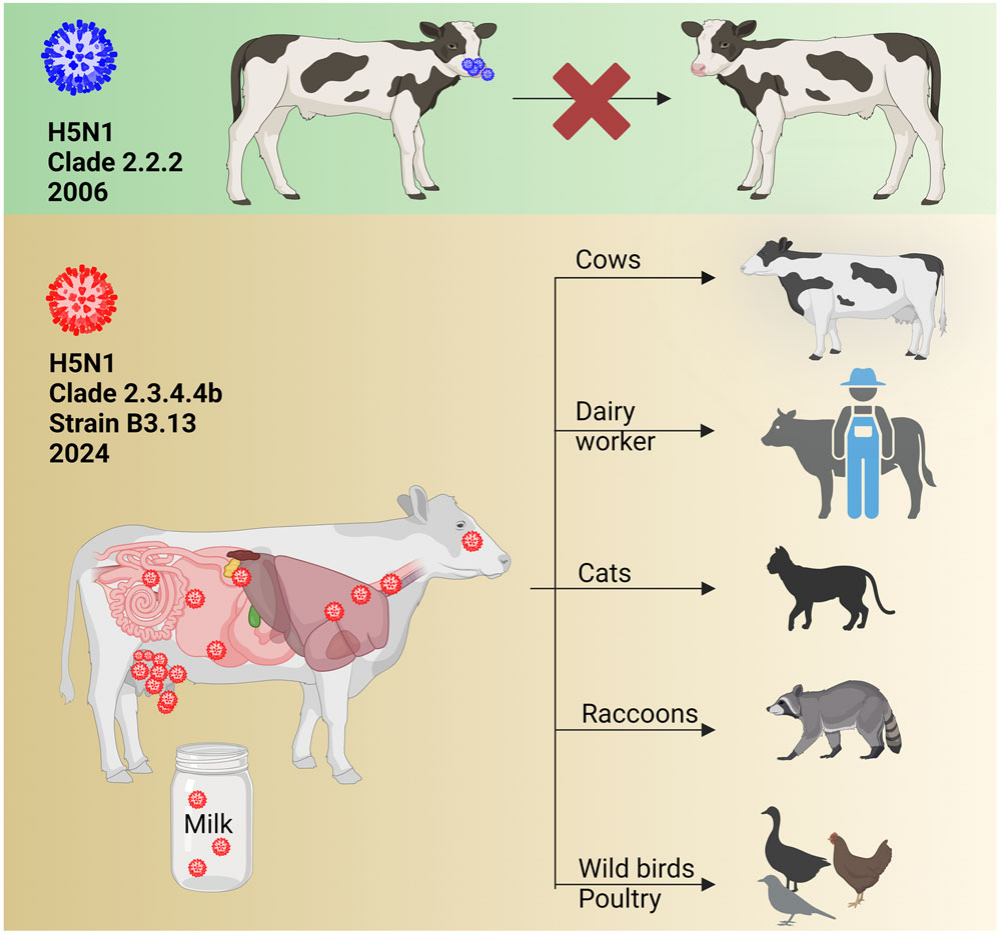
The role of cattle in the transmission of HPAIV H5N1. An experimental infection of calves with an early Gs/Gd isolate of H5N1/2006 clade 2.2.2, isolated from a cat in Germany and with the PB2 mutation E627K, demonstrated inefficient replication and a lack of transmission in calves following intranasal high-titer infection^21^. Conversely, the clade 2.3.4.4b H5N1 B3.13 genotype in the U.S. replicates efficiently in the mammary gland of dairy cows and is capable of transmission to a variety of animals, including wild and domestic birds. The figure was created with BioRender.com.

Previous studies at the Friedrich-Loeffler-Institut, Insel Riems, Germany, showed that a 2006 H5N1 isolate from a cat replicated inefficiently in calves following intranasal high-titer infection^21^. Furthermore, there have been no reports of IAV circulating in cattle, although some spill-over infections of IAV have been described^22^. Therefore, until recently the role of cattle in the epidemiology of IAV was considered insignificant^21^. In contrast, the HPAIV H5N1 clade 2.3.4.4b genotype B3.13 efficiently infected dairy cattle in 2024^23^. Infected cattle may be subclinical or clinical and the main clinical signs are a drastic decrease in milk production, changes in milk consistency, decreased feed uptake, dehydration and fever^23^. The infection is reported to focus on lactating dairy cows and there are very high viral loads in the milk of infected cows. The spread seems to be mainly via the milking process and transport of infected cows to other farms in different states^24^. Apparently, efficient cow-to-cow transmission has allowed the virus to spread to 63 dairy farms in at least 9 states (status as of 05/24/2024). Sequence analysis and phylodynamics suggest a single introduction several months earlier^23,25^. Fatal cases in cats after drinking raw milk from infected cows^24^ and an infection of a dairy farm worker in Texas with a conjunctivitis have been reported^26^. Another study described the transmission of H5N1 from dairy cows to raccoons and to wild and domestic birds^27^ (Fig. 2). In addition, H5N1-RNA has been detected in wastewater^28^, but the source of contamination remains unknown. Importantly, recent research has raised concerns that cattle, once considered a resilient host, may act as a novel IAV host in the future and after further adaptation of H5N1^29^. A recent preprint reports that both avian and human IAV receptors to be present in the upper respiratory tract of cattle and at high densities in bovine udder tissues^29^. These results were used to consider the risk of cows being a “mixing vessel” as known in pigs and fur animals. However, no other IAVs are currently circulating in cattle. Mixing in cattle can therefore be largely ruled out for the time being^15^. Cattle are widely infected with another *Orthomyxovirus* genus, “Influenza D virus (IDV)”. Serological surveillance suggests that IDV can be transmitted to humans, although very rarely compared to AIV^30^. And IDV uses different receptors than IAV^20^.

## Seeking answers to many questions

Influenza A viruses are masters of mutation and adaptation. There are undoubtedly serious questions to be answered about the significant change in the epidemiology of HPAIV H5 from Asia to Europe, the dynamics of infection in wild bird reservoirs, the role of climate change in the spread of the virus, the reasons for the genetic plasticity of the current clade 2.3.4.4b viruses to reassort and adapt to new hosts, and the overall low zoonotic potential of H5 viruses from this clade^31,32^. Vigilance is needed to prevent new waves of infection, especially in mammals, and ultimately to prevent human infection. Dairy cows are the latest and most surprising victims in the list of AIV hosts, but they will not be the last.

Further adaptation of HPAIV H5N1 to cattle must be prevented. Stringent measures such as testing regimes (e.g. via bulk milk diagnostics), strict transport controls, quarantine measures and optimized milking hygiene must be implemented. In addition, much more basic epidemiological data is needed (e.g. seroprevalence on farms, data on non-lactating animals, H5-antibody titers in sera and milk, oro-nasal shedding data). It should also be considered that the most efficient virus shedding cows should be isolated or even euthanized. As a precautionary measure, suitable vaccines should be developed and evaluated, before the virus becomes established in these hosts.
